# Advancing Progressive Web Applications to Leverage Medical Imaging for Visualization of Digital Imaging and Communications in Medicine and Multiplanar Reconstruction: Software Development and Validation Study

**DOI:** 10.2196/63834

**Published:** 2024-12-09

**Authors:** Mohammed A. AboArab, Vassiliki T. Potsika, Alexis Theodorou, Sylvia Vagena, Miltiadis Gravanis, Fragiska Sigala, Dimitrios I. Fotiadis

**Affiliations:** 1 Unit of Medical Technology and Intelligent Information Systems Department of Materials Science and Engineering, University of Ioannina Ioannina Greece; 2 Electronics and Electrical Communication Engineering Department Faculty of Engineering, Tanta University Tanta Egypt; 3 First Propaedeutic Department of Surgery National and Kapodistrian University of Athens Athens Greece; 4 Department of Interventional Radiology General Hospital of Athens Athens Greece; 5 Biomedical Research Institute Foundation for Research and Technology-Hellas, University Campus of Ioannina Ioannina Greece

**Keywords:** medical image visualization, peripheral artery computed tomography imaging, multiplanar reconstruction, progressive web applications

## Abstract

**Background:**

In medical imaging, 3D visualization is vital for displaying volumetric organs, enhancing diagnosis and analysis. Multiplanar reconstruction (MPR) improves visual and diagnostic capabilities by transforming 2D images from computed tomography (CT) and magnetic resonance imaging into 3D representations. Web-based Digital Imaging and Communications in Medicine (DICOM) viewers integrated into picture archiving and communication systems facilitate access to pictures and interaction with remote data. However, the adoption of progressive web applications (PWAs) for web-based DICOM and MPR visualization remains limited. This paper addresses this gap by leveraging PWAs for their offline access and enhanced performance.

**Objective:**

This study aims to evaluate the integration of DICOM and MPR visualization into the web using PWAs, addressing challenges related to cross-platform compatibility, integration capabilities, and high-resolution image reconstruction for medical image visualization.

**Methods:**

Our paper introduces a PWA that uses a modular design for enhancing DICOM and MPR visualization in web-based medical imaging. By integrating React.js and Cornerstone.js, the application offers seamless DICOM image processing, ensures cross-browser compatibility, and delivers a responsive user experience across multiple devices. It uses advanced interpolation techniques to make volume reconstructions more accurate. This makes MPR analysis and visualization better in a web environment, thus promising a substantial advance in medical imaging analysis.

**Results:**

In our approach, the performance of DICOM- and MPR-based PWAs for medical image visualization and reconstruction was evaluated through comprehensive experiments. The application excelled in terms of loading time and volume reconstruction, particularly in Google Chrome, whereas Firefox showed superior performance in viewing slices. This study uses a dataset comprising 22 CT scans of peripheral artery patients to demonstrate the application’s robust performance, with Google Chrome outperforming other browsers in both the local area network and wide area network settings. In addition, the application’s accuracy in MPR reconstructions was validated with an error margin of <0.05 mm and outperformed the state-of-the-art methods by 84% to 98% in loading and volume rendering time.

**Conclusions:**

This paper highlights advancements in DICOM and MPR visualization using PWAs, addressing the gaps in web-based medical imaging. By exploiting PWA features such as offline access and improved performance, we have significantly advanced medical imaging technology, focusing on cross-platform compatibility, integration efficiency, and speed. Our application outperforms existing platforms for handling complex MPR analyses and accurate analysis of medical imaging as validated through peripheral artery CT imaging.

## Introduction

### Background

Vision in 3D is crucial for medical imaging processing and analysis. This technology is critical for displaying volumetric representations of organs, allowing for observation from numerous angles to aid in diagnostic processes, analysis, and decision support [1,2]. Multiplanar reconstruction (MPR) is a technique for 3D medical image visualization in medicine. It reconstructs 3D images from numerous 2D images obtained via modalities such as computed tomography (CT) or magnetic resonance imaging [3-5]. Currently, several medical image processing software packages that provide increased visualization features for medical imaging are available [6,7]. These technologies, which are often housed on desktop computers or workstations with high computing capabilities, offer substantial resources to medical professionals. Concurrently, there has been a marked shift toward developing medical imaging applications and archives that are hosted on the cloud [8-12].

Although web-based medical imaging applications are widespread, there is still a significant gap, as indicated by the unique technological hurdles shown in Table 1. The adoption of progressive web applications (PWAs) in Digital Imaging and Communications in Medicine (DICOM) and MPR visualization is a relatively unexplored area. Our study aimed to address this substantial disparity by examining the incorporation of DICOM and MPR visualization into web settings via PWAs. PWAs possess distinctive characteristics that are relevant to web-based DICOM applications [13-15], such as uninterrupted offline access, enhanced performance, and an enhanced user experience [16,17]. The aim was to address current technology challenges and contribute to the advancement of web-based medical imaging applications for the benefit of radiologists and the health care community.

**Table 1:** Medical imaging visualization on the web—state of the art.

Study	Year	Client technology	Programming type	Functions	Required plug-in
Min et al [18]	2018	Web based	HTML5 and WebGL	2D image processing and 3D visualization	None
Huang et al [6]	2018	Web browser (browser-server structure)	LAMP^a^	Case management module—management of breast ultrasound cases; CAD^b^ subsystem—assisting radiologists in making correct diagnostic decisions	None
Gøeg et al [7]	2018	Web browser	TypeScript, Express framework, and HL7^c^ FHIR^d^ DSTU2^e^	Loose-coupled modules for future-proof telemedicine architecture	None
Herrmann et al [2]	2018	Web browser	Python and C++	Web-based visualization of pathology images; conversion of image files to DICOM^f^ format	None
Zhang [1]	2019	WebGL and JavaScript (Node.js on the server side)	JavaScript (Node.js on the server side)	Web-based medical image rendering and visual synchronization	Not specified
Hazarika et al [12]	2020	Web based using DSpace	JavaScript	Image repository for medical professionals	DWV^g^
Ziegler et al [19]	2020	Web browser (Google Chrome, Firefox, and IE^h^ 11+)	JavaScript	Interactive visualization and analysis of medical images; connectivity to PACSs^i^ via DICOMweb	None
Pemmaraju et al [20]	2022	Web browser	JavaScript (Cornerstone framework for image viewer)	Biomedical image data management and storage—web-based 3D and 4D image viewer for quick evaluation	None
Hazarika et al [21]	2022	DSpace institutional repository	Java and HTML5	Storing and retrieving medical images using DICOM metadata standards	None
Pervan et al [22]	2023	MIDOM^j^ system	Not specified	Efficient and secure exchange of medical images, creation of studies, consultation requests, and responses	Not specified
Bai et al [23]	2023	Platform	JavaScript	DICOM image visualization, annotation and measurement tools, 3D reconstruction, compression and optimization of models, and lung and brain image segmentation	None
Gorman et al [24]	2023	Slim (web-based viewer)	TypeScript and React	Interactive visualization and annotation of slide microscopy images; display of image analysis results	None
Gupta et al [25]	2023	Web browser (Image-IN)	HTML5, JavaScript, VTK.js, and ITK.js	Multidimensional 3D visualization (volume, surface, and triplanar and multiplanar rendering)	None
Chen et al [26]	2023	BlueLight	JavaScript and HTML5	Lightweight pure single-page application for medical image interpretation and 3D image reconstruction (MPR^k^, MIP^l^, and VR^m^)	None

^a^LAMP: Linux+Apache+MySQL+PHP (hypertext preprocessor).

^b^CAD: computer-aided diagnosis.

^c^HL7: Health Level Seven.

^d^FHIR: Fast Healthcare Interoperability Resources.

^e^DSTU2: Draft Standard for Trial Use 2.

^f^DICOM: Digital Imaging and Communications in Medicine.

^g^DWV: DICOM Web Viewer.

^h^IE: Internet Explorer.

^i^PACS: picture archiving and communication system.

^j^MIDOM: Medical Imaging and Diagnostics on the Move.

^k^MPR: multiplanar reconstruction.

^l^MIP: maximum intensity projection.

^m^VR: volume rendering.

Another significant challenge is ensuring that these technologies are seamlessly integrated into existing health care systems. Essential considerations include cross-platform compatibility, integration capabilities, speed, and scalability [27,28]. A third challenge is the MPR for medical image visualization, which involves generating high-resolution images on the web and viewing volumetric structures, more specifically, the coronal and sagittal views obtained from DICOM slices [26]. The complexities of MPR require careful examination, and our research strove to clarify and address these issues to enhance web-based medical imaging. In the field of web-based medical imaging visualization, Min et al [18] investigated the use of HTML5 and WebGL to overcome difficulties associated with remote accessibility for radiologists. Their main objective was to create a CT colonography demonstration and assess its performance on different browsers and operating systems. The findings of their study suggest that HTML5 and WebGL have the potential to enhance remote access to medical imaging, although some modest limits and browser compatibility concerns were recognized. The research conducted by Hazarika et al [12,21] focused on the development of DICOM-based medical image repositories using DSpace. The objective of their work was to improve the accessibility of medical images and minimize the expenses associated with storage. Although the indexing system effectively enables the accommodation and retrieval of DICOM images, it faces scalability constraints when dealing with larger datasets. The practical ramifications entail the creation of a web-based platform for DICOM users, which offers affordable storage expenses. Wadali et al [29] assessed open-source alternatives, including eSanjeevani, the Indian National Telemedicine Service, within the domain of web-based DICOM viewers. Their study recommends the use of the DICOM Web Viewer because of its notable features and compatibility. It acknowledges both the merits and shortcomings of the DICOM Web Viewer while underlining the importance of a tailored solution. The limitations of eSanjeevani include the lack of a comprehensive viewer and the need for further development of a picture archiving and communication system (PACS) server to improve teleradiology efficiency. Gorman et al [24] provided Slim, a freely available microscope slide viewer that adheres to the DICOM standard and is designed for use with the National Cancer Institute Imaging Data Commons. The authors highlight the practical usefulness of DICOM in quantitative tissue imaging. The study admits limits in standardization, interoperability, and data formats. The Open Health Imaging Foundation Viewer developed by Ziegler et al [19] is a versatile web-based medical image viewer used in the field of cancer research. Although their paper recognizes the widespread adoption and comprehensive functionality of the system, it also describes certain limits. These limitations include difficulties in implementing the system for users who are not technically inclined, highlighting the importance of raising community awareness and providing thorough documentation. Jodogne [30] introduced VolView, an open-source web-based DICOM viewer offering cinematic volume rendering for medical professionals. While it enhances accessibility to volumetric imaging, tool deployment may be challenging for nontechnical users, and its reliance on integration with specific PACSs could limit broader adoption. Lebre et al [31] introduced “Dicoogle,” an open-source PACS archive designed to address the challenges of managing large-scale medical imaging data. Dicoogle’s flexible, plug-in–based architecture enables easy extension and customization, making it suitable for research, education, and clinical environments. However, despite its robust capabilities, Dicoogle’s reliance on user-developed plug-ins for specific functionalities may limit its out-of-the-box utility. Chen et al [26] developed Blue Light, an open-source DICOM viewer that uses cost-effective calculation methods in JavaScript for web-based medical imaging. This web-based reader, designed for mobile devices, focuses on reducing computation time and memory use. It prioritizes stability and speed. Their paper acknowledges recommendations for enhancing rendering speed, specifically in mobile apps, and highlights the efficacy of BlueLight in rendering 3D medical pictures with DICOM annotations. Ghoshal et al [32] proposed an approach for reconstructing 3D spine magnetic resonance imaging using bicubic interpolation in MPR and 3D visualization. Although the study demonstrates excellent precision, it does not include direct comparisons, exhibits potential performance discrepancies within datasets, and concentrates on specific circumstances. Fajar et al [33] provide a technique that effectively handles metadata variations in DICOM files by reconstructing and resizing 3D images. This algorithm is particularly useful for efficiently managing big data. Although the study made substantial contributions to the production of 3D images, it does not include direct comparisons with existing approaches. Furthermore, additional research is needed to overcome potential difficulties in handling various DICOM files.

### Objectives

Our study investigated the integration of DICOM and MPR visualization into web environments through PWAs, with the goal of overcoming current technology disparities and improving web-based medical imaging functionalities. By using the distinctive attributes of PWAs, including seamless offline access and improved performance, our study endeavored to offer a holistic solution to address challenges such as cross-platform compatibility, integration capabilities, speed, and scalability. The ultimate aim was to benefit radiologists and the health care community, particularly in addressing issues related to peripheral artery disease. The code of this work is available on GitHub [34].

## Methods

The architectural framework and design features of our DICOM and MPR web visualizations are presented in the following sections.

### Application Design

**Figure 1:**
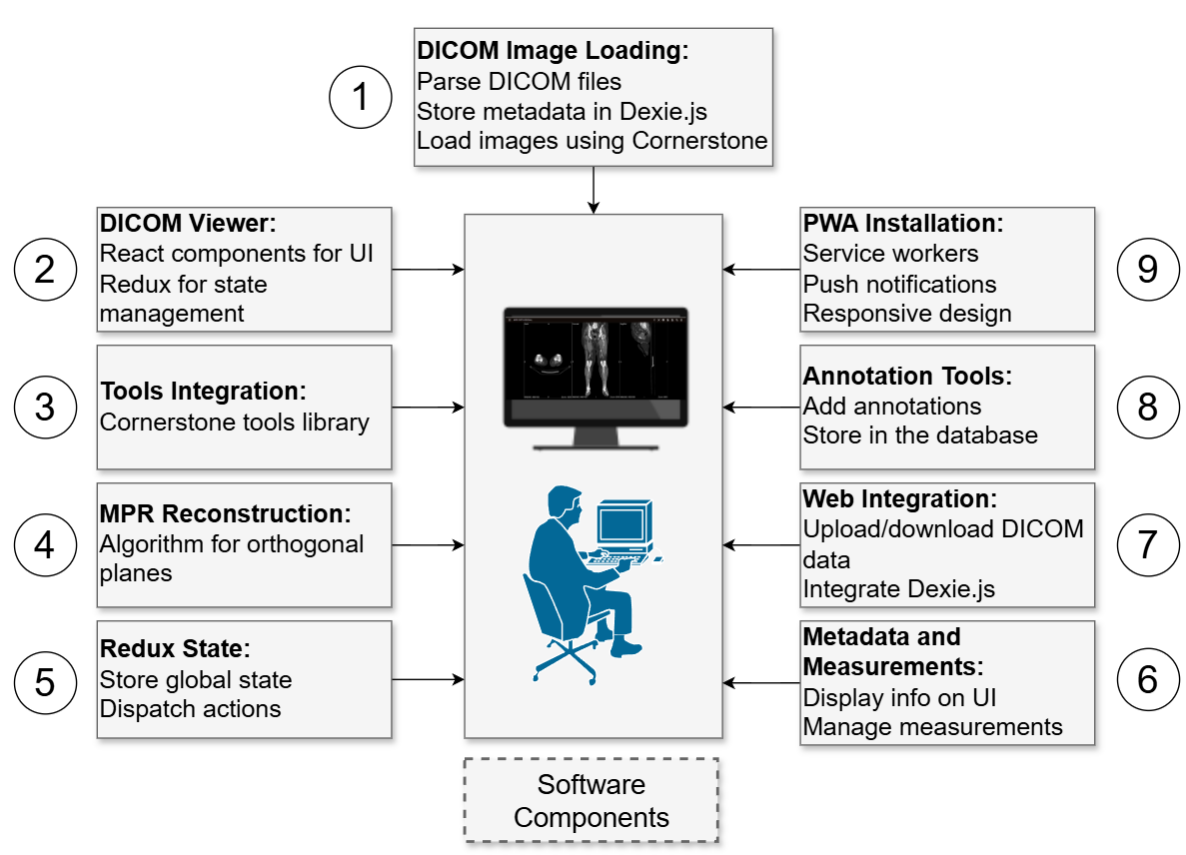
Pipeline architecture of Digital Imaging and Communications in Medicine (DICOM) and multiplanar reconstruction (MPR) visualization as a progressive web application (PWA). UI: user interface.

The architectural framework of the DICOM and MPR PWA was designed with modular components to provide a seamless user experience, enabling a smooth transition from data upload to visualization. Metadata and image information play pivotal roles in retrieving crucial details such as transfer syntax, service-object pair classes and instances [23,35], pixel representations, planar configurations, viewer elements, and image loading. As shown in Figure 1, the design encompasses several key modules that work together to facilitate efficient DICOM image handling and MPR. The DICOM image loading (step 1) module is responsible for parsing DICOM files via the Cornerstone library, which includes the dicomParser tool. This process extracts metadata and image data from DICOM files, storing them locally in Dexie.js to ensure that the images are prepared and ready for visualization and manipulation within the application. In contrast, the web integration (step 7) module focuses on integrating DICOM data with the broader web environment. This includes functionalities for uploading and downloading DICOM datasets, allowing users to interact with remote DICOM stores. Dexie.js manages the storage and retrieval of data during these interactions. The key distinction between steps 1 and 7 lies in their scope—step 1 handles the initial parsing and local loading of DICOM images, whereas step 7 manages the integration of these images into a web-based workflow, encompassing data transfer operations between the client and server. The DICOM viewer (step 2), tool integration (step 3), and MPR (step 4) modules further enhance the application by providing React.js components for the user interface, integrating Cornerstone tools for image manipulation, and implementing algorithms for orthogonal plane reconstructions, respectively. Both frameworks, Cornerstone.js and React.js, improve performance but face limitations particularly with complex state management and ensuring cross-platform compatibility. The Redux state (step 5) module is used for global state management, ensuring consistent state handling across the application. Additional features include metadata and measurements (step 6), which manage the display of crucial image information and measurements, and PWA installation (step 9), which enables offline access and enhances performance.

The DICOM web application integration functions play an instrumental role in establishing connectivity to the DICOM store, facilitating the search and loading of specific studies, and retrieving DICOM instances for detailed analysis. Leveraging the PWA approach, seamless DICOM store connectivity; focused study examination; and web-based viewer controls for zooming, moving or panning, and resetting are ensured by the application. The functionality of reference lines and planes is tailored to create reference lines for aligning and comparing DICOM images within the MPR views [36]. This involves the construction of 3D lines and planes to represent spatial relationships, as well as the coordination of transformations for converting 3D perspectives into 2D images. DICOM viewer rendering within the PWA is achieved through the setting of DICOM image references, handling image clicks, identifying localizer images, and synchronizing DICOM slices [20,22].

### MPR Algorithm

#### Overview

In the MPR from CT DICOM images, the integration of bicubic interpolation and weighted bilinear interpolation plays a significant role in improving the accuracy and visual fidelity of the reconstructed volumes. Bicubic interpolation, which uses a smooth and differentiable interpolation function across a 4 × 4 grid, proves valuable in addressing edge cases and delivering high-quality reconstructions [37]. The overall interpolation process benefits from the straightforward cubic interpolation method, which contributes to computational efficiency while maintaining a satisfactory level of smoothness in the interpolated values [38]. In addition, a balance between simplicity and effectiveness is achieved through the application of weighted bilinear interpolation for nonedge pixels. This technique combines the weighted contributions of neighboring pixels, facilitating the generation of interpolated values, which are crucial in constructing detailed and coherent representations of volume from DICOM images. The integration of these interpolation methods enhances the robustness and accuracy of the MPR, ultimately improving the diagnostic capabilities of the reconstructed volumetric data in the field of peripheral arterial diagnosis.

#### Weighted Bilinear Interpolation for Nonedge Pixels

Weighted bilinear interpolation is applied to nonedge pixels for the generation of interpolated planes between original planes in a volumetric dataset. Given an interpolation weight *w* derived from the relative position of the target pixel within the interpolation interval, the value of an interpolated pixel *P*(*k*) at position *k* is calculated via a weighted average of its neighboring pixels in the original planes [39]. The interpolation considers the direct neighbor pixels along the same axis in both the current and the next interval planes. The weighting factors for the neighbors are adjusted to account for the distance from the interpolated position, emphasizing closer neighbors more significantly. For a nonedge pixel *k* located at a position in which *k* – 1 > 0 and *k* + 1 < length, the interpolated pixel value *P*(*k*) is given as follows [40,41]:

*P*(*k*) = *P_i_*(*k*) + *P_i_*_+1_(*k*) (**1**)

*P_i_*(*k*) = (*V_i_*[*k*] × [1 – *w*] × 0.5 + *V_i_*[*k* – 1] × [1 – *w*] × 0.25 + *V_i_*[*k* + 1] × [1 – *w*] × 0.25) (**2**)

*P_i_*_+1_(*k*) = (*V_i_*_+1_[*k*] × *w* × 0.5 + *V_i_*_+1_[*k* – 1] × *w* × 0.25 + *V_i_*_+1_[*k* + 1] × *w* × 0.25) (**3**)

In these equations, *V_i_*[*k*] denotes the value of pixel *k* in the original plane at interval *i*, and *V_i_*_+1_[*k*] is the value of pixel *k* in the next original plane at interval *i* + 1. For edge cases, where *k* – 1 < 0 or *k* + 1 ≥ length, the interpolation simplifies the prioritization of available neighboring pixels, reducing the weighting factors to 0.75 for the pixel itself and 0.25 for the available neighbor, accordingly adjusted through the interpolation weight.

#### Bicubic Interpolation

Bicubic interpolation extends cubic interpolation to 2 dimensions, providing a smooth and continuous interpolation function. Given a 4 × 4 grid of data points *P_ij_* and 2 interpolation parameters *u* and *v*, the bicubic interpolation equation is as follows [37,42]:


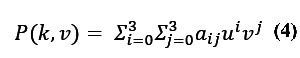


The coefficients *a_ij_* are determined on the basis of the values of *P_ij_* and their partial derivatives. Bicubic interpolation is commonly used for reconstructing detailed and high-resolution images from CT DICOM slices. These interpolation methods are fundamental in the reconstruction process, contributing to the accurate representation of CT DICOM images in MPRs.

**Figure 2:**
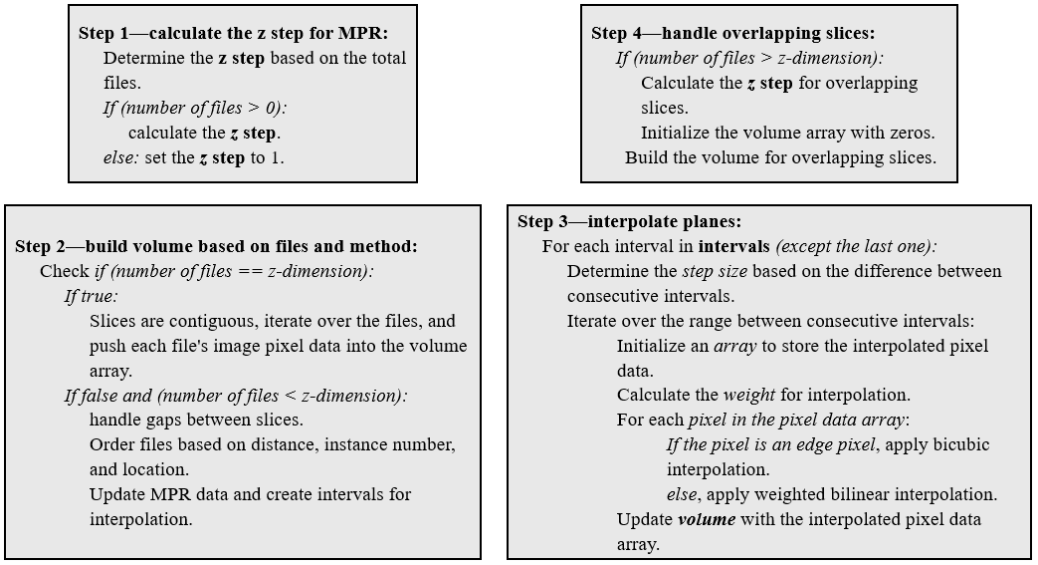
Multiplanar reconstruction (MPR) protocol for volumetric data from Digital Imaging and Communications in Medicine slices.

The MPR protocol is structured as shown in Figure 2 to ensure comprehensive reconstruction of volumetric data from DICOM slices [26]. The design involves calculating the *z* step for the MPR, building a volume based on files and method specifications, interpolating planes, and handling overlapping slices. In the calculation of the *z* step for the MPR, the protocol determines the *z* step based on the total files and specified MPR dimensions, handling cases in which the number of files is 0. The volume is contingent on whether the number of files matches the specified *z*-dimension. The protocol processes contiguous slices and handles gaps between slices, ordering files based on distance, instance number, and location. Interpolating planes involves determining step sizes, iterating over intervals, and applying bicubic or weighted bilinear interpolation based on pixel characteristics. The protocol addresses overlapping slices by calculating the *z* step for overlapping slices and selectively building the volume. This comprehensive protocol ensures effective MPR, facilitating nuanced analysis of volumetric data from DICOM slices in medical imaging.

### Application Implementation

#### Technology Stack

The DICOM and MPR web application is implemented as a PWA, leveraging the capabilities of React.js and Cornerstone.js [43].

#### PWA Implementation

PWAs offer a seamless and responsive user experience across various devices and platforms. DICOM and MPR web applications use the power of PWA, ensuring accessibility to different browsers and providing users with the ability to install the application on their devices [17].

#### Front-End Framework

React serves as the foundational front-end library for DICOM and MPR web implementation. Its component-based architecture facilitates the modular design of the application, enabling efficient updates and rendering of DICOM images. The React declarative approach enhances the predictability of the user interface, contributing to a smooth user experience [44].

#### DICOM Image Processing

Cornerstone.js, a robust JavaScript library designed for medical imaging, plays a pivotal role in DICOM image rendering and analysis within the proposed application. Cornerstone.js seamlessly integrates with React, providing a suite of tools tailored for DICOM image analysis, as shown in Textbox 1.

**Digital Imaging and Communications in Medicine (DICOM) image annotation and measurement tools and their functions.**

- Length tool: allows for the measurement of distances on DICOM images
- Pan tool: enables users to pan across DICOM images for detailed examination
- Magnify tool: provides magnification capabilities for closer inspection of image details
- Angle tool: facilitates angle measurements for anatomical analysis
- Rectangle region-of-interest (ROI) tool: allows for the creation of rectangular ROIs on DICOM images
- Window width and window center tool: enables adjustment of the window width and window center for optimal image visualization
- Zoom touch pinch tool: supports touch-based zooming gestures for enhanced user interaction
- Probe tool: provides pixel value information at specific points on DICOM images
- Elliptical ROI tool: allows for the creation of elliptical ROIs for focused analysis
- Freehand ROI tool: enables the creation of freehand ROIs on DICOM images
- Stack scroll mouse wheel tool: facilitates smooth scrolling through DICOM image stacks, enhancing the user’s ability to navigate volumetric data

#### Data Storage

Dexie is a JavaScript library that simplifies interaction with IndexedDB. IndexedDB is used as a low-level data storage application programming interface, enabling the storage and retrieval of large amounts of data in the browser. Dexie provides a concise application programming interface that streamlines data management processes, contributing to a more intuitive developer experience [45].

#### Cross-Browser Compatibility and Platform Accessibility

DICOM and MPR visualization, built using PWA principles, ensure cross-browser compatibility, making the application accessible on various browsers, including Google Chrome, Firefox, Safari, and Microsoft Edge. The application’s responsive design guarantees optimal performance across different platforms, including desktops, tablets, and mobile devices [46].

The integration of React, Cornerstone.js, and Dexie empowers the development of the DICOM- and MPR-based PWA with a robust foundation, advanced DICOM image analysis tools, and efficient data management capabilities. The adherence to PWA principles further extends the application’s accessibility and user engagement across diverse environments.

### Ethical Considerations

The National and Kapodistrian University of Athens provided a dataset for the experiments, comprising CT scans from 22 patients diagnosed with peripheral artery disease. The data were collected via a Revolution EVO CT scanner and were randomly selected and anonymized from the hospital system for ethical reasons. The research protocol was approved under protocol numbers 9876/28.3.24 and 11293/9.4.24.

## Results

### Overview

Detailed findings from 2 experiments on the DICOM-based PWA for medical image visualization and reconstruction are provided in this section. The key aspects include performance evaluation across platforms, dataset characteristics, computer specifications, and testing metrics. The experimental findings highlight various browser performances on different platforms for loading, volume rendering, and tool execution in local area network (LAN) and wide area network (WAN) environments, emphasizing Chrome’s (Google LLC) superiority in loading and rendering, whereas Firefox (Mozilla Foundation) excelled in viewing slices.

### Experimental Design

In the experimental design phase, our application assisted radiologists in connecting to upload medical images seamlessly from local devices. The upload options included single files, folders, or links containing DICOM images. Once uploaded, our application provided a variety of essential tools for data access, annotation and measurements, image processing, and MPR, ensuring a comprehensive environment for image manipulation within a web browser. To assess the effectiveness of the DICOM- and MPR-based PWA for medical image visualization and reconstruction, 2 distinct experiments were performed. The first experiment aimed to gauge the application’s performance across multiple platforms, ensuring compatibility and optimal functionality.

The second experiment was designed to evaluate the application’s performance under controlled network conditions. Specifically, tests were conducted within an LAN to minimize the effect of internet variability, ensuring consistent bandwidth and reduced latency. This approach provided a stable environment for accurately assessing the software’s inherent performance, whereas comparisons were also made under WAN settings to understand the impact of broader network conditions.

The dataset provided by the University of Athens was used to evaluate the performance of this work on different platforms and browsers. Each dataset series is characterized by its unique dimensions, which vary between 512 × 512 × 258 pixels and 512 × 512 × 577 pixels and represent the width, height, and depth of the CT scans. The dataset series sizes range from 128 to 290 MB. In addition, the dataset exhibits variability in slice thickness, spacing between slices, and pixel spacing, with values of 5.0, 0.976562, and 0.775391 mm, respectively. These details elucidate the characteristics of the dataset, providing a comprehensive understanding of its diversity, which is crucial for the thorough evaluation of the work across different platforms and browsers. A comprehensive overview of the dataset series is presented in Table 2, whereas details about the computers used in the experiments are shown in Table 3. Notably, the computers used were standard laptops accessible to regular users. React and PWAs are supported by all major browsers, including Firefox (version 125.0.3), Google Chrome (version 125.0.6422.78), Safari (Apple Inc), Internet Explorer (Microsoft Corp), and Microsoft Edge (version 125.0.2535.67; Microsoft Corp).

**Table 2:** Characteristics of the computed tomography dataset for peripheral artery patients used in evaluation.

Patient	Dimensions (pixels)	Size (MB)	Slice thickness (mm)	Spacing between slices (mm)	Pixel spacing (mm)
1	512 × 512 × 377	189	3.75	3.75	0.935547
2	512 × 512 × 274	138	3.75	3.75	0.960938
3	512 × 512 × 386	194	3.75	3.75	0.976562
4	512 × 512 × 531	267	2.5	2.5	0.841797
5	512 × 512 × 384	193	3.75	3.75	0.955078
6	512 × 512 × 258	130	5.0	0	0.8984375
7	512 × 512 × 577	290	2.5	2.5	0.912109
8	512 × 512 × 334	168	3.75	3.75	0.976562
9	512 × 512 × 350	176	3.75	3.75	0.976562
10	512 × 512 × 340	171	3.75	3.75	0.949219
11	512 × 512 × 352	177	3.75	3.75	0.888672
12	512 × 512 × 377	189	3.75	3.75	0.976562
13	512 × 512 × 277	139	5.0	5.0	0.976562
14	512 × 512 × 310	156	3.75	3.75	0.925781
15	512 × 512 × 255	128	5.0	5.0	0.976562
16	512 × 512 × 346	174	3.75	3.75	0.925781
17	512 × 512 × 374	188	3.75	3.75	0.902344
18	512 × 512 × 269	135	0.625	5.0	0.976562
19	512 × 512 × 298	150	3.75	3.75	0.939453
20	512 × 512 × 316	159	3.75	3.75	0.976562
21	512 × 512 × 341	171	3.75	3.75	0.896484
22	512 × 512 × 310	156	3.75	3.75	0.775391

**Table 3:** Specifications of the computers used in the experiments.

Computer	Type	Operating system	CPU^a^	Memory (GB)	GPU^b^
1	Laptop	Windows 11 Pro 64 bits	11th-generation Intel Core i7-11800H at 2.30 GHz; 16 cores	16	NVIDIA GeForce RTX 3070
2	Laptop	Ubuntu 22.04.3 LTS^c^	11th-generation Intel Core i7-11800H at 2.30 GHz; 16 cores	16	NVIDIA GeForce RTX 3070
3	Laptop	macOS 14 Sonoma	11th-generation Intel Core i7-11800H at 2.30 GHz; 4 cores	8	NVIDIA GeForce RTX 3070
4	Tablet	Android 5.0.2 (Lollipop)	Quad-core 1.2-GHz Cortex-A7	1.5	Adreno 305

^a^CPU: central processing unit.

^b^GPU: graphics processing unit.

^c^LTS: long-term support.

The performance evaluation of the proposed system included several key metrics, as detailed in Table 4, essential for ensuring its clinical viability. T1 represents the performance time for loading a medical image dataset, assessing the time required to load an entire DICOM dataset into the application. This metric was selected due to the critical need for rapid image access in clinical settings, where delays could hinder diagnostic workflow efficiency. T2 evaluates the performance time to build a medical image volume using the MPR technique, reflecting the time necessary to reconstruct 3D volumes from 2D slices. This is crucial for providing clinicians with timely and accurate 3D representations, which are often essential for diagnostic and surgical planning. T3 monitors the performance time for viewing a slice while scrolling through a medical image dataset, a vital metric for ensuring that radiologists can efficiently navigate through large datasets to identify relevant anatomical structures. T4 focuses on the performance time for annotation and measurement tools per slice, which include tools such as “Wwwc,” “Pan,” and “Zoom” and region-of-interest tools. The efficiency of these tools is directly linked to the accuracy and speed of clinical assessments. Finally, T5 measures the performance time for image processing tools, specifically the invert tool per slice, which is essential for enhancing contrast and improving the visibility of subtle pathologies, thus aiding in more accurate diagnoses. By minimizing wait times and enabling faster decision-making, these metrics directly correlate with the clinical efficiency and reliability of the application, ensuring that it meets the demands of medical professionals in real-world settings.

**Table 4:** Performance metrics details.

Function and label		Description	Measurement
**Data access**			
	T1	Performance time for loading a medical image dataset	Measured using JavaScript code
	T2	Performance time to build a medical image volume using MPR^a^ techniques	Measured using JavaScript code
	T3	Performance time for viewing a slice in a medical image dataset while scrolling	Measured using JavaScript code
**Annotation and measurement tools**			
	T4	Performance time for the following tools: “Wwwc,” “Pan,” “Zoom,” “Length,” “Probe,” “EllipticalRoi,” “RectangleRoi,” “Angle,” “Magnify,” and “FreehandRoi” per slice	Measured using JavaScript code
**Image processing**			
	T5	Performance time for invert tool per slice	Measured using JavaScript code

^a^MPR: multiplanar reconstruction.

### Performance Across Multiple Platforms

#### Overview

In the initial experiment, we used a dataset of 22 patients via LAN to assess the application’s performance on various platforms. The demonstration of our application underwent testing on computers running Windows, Linux, and macOS, executing each function 5 times with different browsers, and the averages were calculated. Table 5 presents the average performance for each function across the entire dataset of 22 patients.

**Table 5:** Performance metrics for the proposed application across platforms, browsers, and modes (private and ordinary).

Platform		Windows					Linux			macOS		
		Google Chrome (s)	Microsoft Edge (s)	Firefox (s)	Internet Explorer (s)	Google Chrome (s)		Firefox (s)	Google Chrome (s)		Firefox (s)	Safari (s)
**Private**												
	AT^a^-T1^b^ (LAN^c^)	0.778	0.826	0.874	0.958	—^d^		—	—		—	—
	AT-T2^d^ (LAN)	5.157	5.03	6.306	5.276	—		—	—		—	—
**Ordinary**												
	AT-T1 (LAN)	1.036	1.22	0.89	1.305	1.101		6.45	4.434		5.736	6.443
	AT-T2 (LAN)	5.215	5.344	6.33	5.277	5.06		6.664	7.69		9.26	19.559
	AT-T1 (WAN^e^)	1.212	1.119	0.842	1.263	1.412		5.914	4.823		5.388	5.943
	AT-T2 (WAN)	5.068	5.202	6.478	5.39	5.175		6.277	7.276		9.057	19.6
	AT-T3^f^ (WAN)	0.00175	0.00188	0.0014	0.0019	0.00177		0.00168	0.00217		0.0015	0.00357
	AT-T4^g^ (WAN)	0.000155	0.00013	0.0005	0.00015	0.000135		0.0005	0.000155		0.0012	0.0006

^a^AT: average time.

^b^T1: performance time for loading a medical image dataset.

^c^LAN: local area network.

^d^Not measured.

^d^T2: performance time to build a medical image volume using multiplanar reconstruction techniques.

^e^WAN: wide area network.

^f^T3: performance time for viewing a slice in a medical image dataset while scrolling.

^g^T4: performance time for the following tools: “Wwwc,” “Pan,” “Zoom,” “Length,” “Probe,” “EllipticalRoi,” “RectangleRoi,” “Angle,” “Magnify,” and “FreehandRoi” per slice.

#### Private Mode Impact on Loading (T1) and Volume Rendering (T2)

The private mode generally contributed to faster loading times (T1) across browsers. The impact on volume rendering times (T2) varied, with some browsers showing minor improvements in private mode. These findings provide insights for users seeking optimal performance during medical image visualization and reconstruction.

#### Performance Evaluation on Windows, Linux, and macOS on LAN and WAN

Regarding T1 loading, Google Chrome demonstrated superior performance on Windows (1.036 seconds), Linux (1.101 seconds), and macOS (4.434 seconds), whereas Firefox showed a competitive performance on Windows at 0.89 seconds. Regarding T2 volume rendering, Google Chrome consistently outperformed other browsers on all platforms, with the shortest times on Windows (5.215 seconds), Linux (5.06 seconds), and macOS (7.69 seconds). Firefox demonstrated a competitive time of 6.33-second viewing slices on Windows in a medical image dataset while scrolling (T3); Firefox outperformed other browsers across all platforms in scrolling performance within the medical image dataset (T3), achieving the fastest times on Windows (0.0014 seconds), Linux (0.00168 seconds), and Mac (0.0015 seconds).

Regarding T4 tool performance per slice, Google Chrome and Microsoft Edge on Windows exhibited the fastest times at 0.000155 and 0.00013 seconds, respectively. Google Chrome on Linux and macOS demonstrated efficiencies of 0.000135 and 0.000155 seconds, respectively. Google Chrome exhibited superior performance for T1 loading and T2 volume rendering, whereas Firefox outperformed in T3, and Google Chrome or Microsoft Edge led in the execution of T4 tools per slice across different platforms in an LAN environment. In a WAN environment, for T1 loading, Google Chrome exhibited an efficient performance across all platforms, with the shortest times on Windows (1.212 seconds), Linux (1.412 seconds), and macOS (4.823 seconds). Firefox showed a competitive performance on Windows with 0.842 seconds. Regarding T2 volume rendering, Google Chrome consistently outperformed other browsers on all platforms, with the shortest times on Windows (5.068 seconds), Linux (5.175 seconds), and macOS (7.276 seconds).

Google Chrome demonstrated superior performance for both T1 loading and T2 volume rendering across Windows, Linux, and macOS platforms in a WAN environment.

The experiments demonstrated that the performance time for 2D image processing, specifically for the inverted tool per slice (T5), consistently remained significantly at <1 second across all the computers. This finding leads to the conclusion that the application exhibits real-time performance capabilities for all the provided 2D tools, indicating its efficiency and responsiveness in handling the DICOM peripheral artery dataset.

### Qualitative and Quantitative Evaluation

The proposed application, which focuses on the accuracy of MPR for both coronal and sagittal views, was evaluated by a board-certified medical doctor and surgeon with experience in the evaluation of CT images. This assessment used both qualitative and quantitative methods to ensure a comprehensive analysis of the application’s performance in medicine through the random selection of some dataset series, as shown in Table 2. The measurements were conducted manually via the tools of our application. Bone structures were selected for measurements because of the high edge contrast of bones, which provides clearly visible edges for placing measurement points. Care was taken to ensure that the measurements corresponded to the same structure, position, and plane across all 3 views: axial, coronal, and sagittal. In the axial view, which represents the data source, the structure is measured along 2 axes that represent the coronal and sagittal planes of that structure and are compared with the measurements of the same structure in the reconstructed coronal and sagittal planes. The measurement points were placed on the edges of the structures using the mouse cursor in a magnified view of the structure, ensuring that the pixels representing the exact edge were selected.

**Figure 3:**
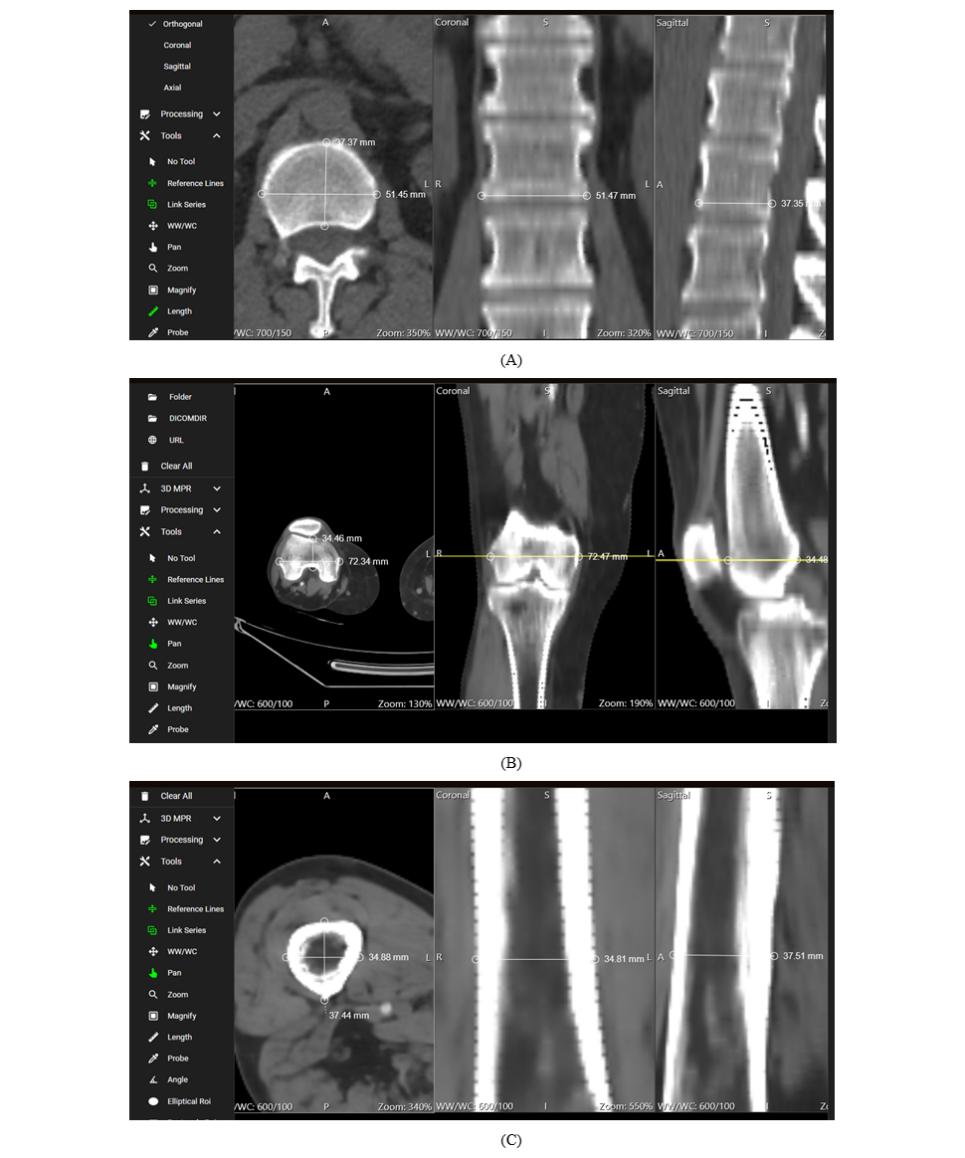
Clinical evaluation of multiplanar reconstruction accuracy—(A) measurement accuracy of the distal edge of the L1 vertebra in the dataset series of patient 1, (B) consistency in measuring the distal head of the femur bone in the dataset series of patient 2, and (C) consistency of reference lines across multiple planes for the dataset series of patient 3.

For the dataset series of patient 1, the distal edge of the L1 vertebra was chosen as a measurement point. For the dataset series of patient 2, the measurement focused on the distal head of the femur bone. Measurements for the dataset series of patients 3, 4, 10, and 20 were performed on the body of the femur bone. The results demonstrated consistent accuracy in the measurements of the reconstructions compared with the ground-truth images across all the examined datasets, as shown in Figure 3. The error margin was computed by comparing the coronal and sagittal measurements (measured values; *M*) to the axial view (ground truth; *G*). The error for each measurement was calculated as follows:


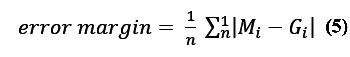


In this equation, *n* represents the total number of measurements. *M_i_* refers to the *i* – *th* measured value (in the coronal or sagittal view), and *G_i_* refers to the corresponding ground-truth value (from the axial view) at the same measurement point. The subscript *i* indexes each measurement point, running from 1 to *n*. This formula provides the average error per measurement. Across the dataset, the measurements consistently fell within an accepted error margin of <0.05 mm, which was primarily attributed to the inherent limitations of manual measurement methods, such as the placement of measurement points via the mouse cursor.

To further assess the accuracy of the MPR reconstructions, a comparative analysis was conducted using 3D Slicer (version 5.6.2; the Slicer Community) [47] on the same dataset series of patient 1, as shown in Multimedia Appendix 1. The distal edge of the L1 vertebra was identified across all planes (axial, coronal, and sagittal) via a similar methodology. Measurements were performed using the native tool in 3D Slicer, where the markers were manually placed using the mouse pointer. Measurements were first taken in the axial plane and then repeated in the coronal and sagittal planes. The measurements in the axial plane via 3D Slicer were nearly identical to those obtained via the proposed PWA. Similarly, the measurements in the coronal and sagittal planes were consistent with both the axial plane results and the measurements obtained from the proposed PWA. As in the proposed PWA, the manual process of placing the cursor at the perceived edge of the structure introduced small variations (<0.5 mm) between measurements. These differences were attributed to the sensitivity of the mouse cursor positioning and the inherent limitations of manual measurement. The consistent appearance of this error margin in both the proposed PWA and 3D Slicer indicates that it is due to the manual measurement process.

The findings of the evaluation indicate the ability of our application to deliver both qualitative and quantitative benefits in medicine. By offering precise measurements and consistent reference lines across various planes, the application represents a valuable tool for medical professionals.

## Discussion

### Principal Findings

The key contribution of this study lies in addressing the gap in the adoption of PWAs for DICOM and MPR visualization on the web. This study highlights the unique challenges in web-based medical imaging, such as cross-platform compatibility, integration capabilities, speed, and scalability. By specifically focusing on the incorporation of DICOM visualization into web settings via PWAs, this study aimed to provide a comprehensive and effective solution to enhance the functionality and efficacy of medical imaging applications in the digital era.

A significant finding of our study is the varying performance of the PWA across different browsers and platforms, which has direct implications for its deployment in clinical settings. Google Chrome outperformed other browsers in terms of loading times (T1) and volume reconstruction efficiency (T2), particularly on Windows and Linux, owing to its efficient V8 JavaScript engine. Firefox demonstrated a strong performance in slice scrolling (T3) but exhibited slower (T2) performance on macOS, likely due to differences in memory management. Safari and Microsoft Edge lagged behind in T1 and T2, especially on macOS, with Safari showing the slowest performance.

These observations underscore the challenges of ensuring cross-platform consistency, with macOS generally showing a slower performance, particularly for T2. Given these findings, Google Chrome or Firefox on Windows is recommended for optimal performance, particularly in environments requiring rapid data access and processing. This study also highlights the importance of selecting the appropriate browser based on the specific clinical setting as performance can vary significantly depending on the browser and platform used. This insight is critical for health care providers aiming to implement PWAs in their medical imaging workflows.

Furthermore, this study emphasizes the significance of uninterrupted offline access, enhanced performance, and improved user experience as distinctive characteristics of PWAs relevant to web-based DICOM applications. By doing so, this study aimed to overcome the identified technological hurdles and contribute to the advancement of web-based medical imaging applications. The choice of DICOM as the focus further solidifies the relevance of this study in the medical imaging domain, where standardization and interoperability are crucial.

In addition, this study contributes to the literature by addressing another challenge in the field of medical imaging applications, namely, the lack of an effective method for addressing variables inherent to web applications. The emphasis on cross-platform compatibility, integration capabilities, speed, and scalability underscores the commitment to providing a holistic solution that goes beyond DICOM and MPR visualization.

The discussion on MPR for medical image visualization adds depth to the contribution. The challenges related to generating high-resolution images on the internet and visualizing volumetric structures, especially sagittal and coronal views obtained from DICOM slices, are acknowledged. This research strives to clarify and address these issues, aiming to enhance web-based medical imaging capabilities, particularly in the field of peripheral artery imaging.

The MPR algorithm proposed in this study used bicubic and weighted bilinear interpolation, which enhances edge detail, particularly in scenarios in which certain resolutions may result in missing intermediate points. This approach differs from conventional implementations such as XTK.js and VTK.js [30], which primarily use linear interpolation to prioritize computational efficiency. XTK.js adopts linear interpolation to balance smoothness and performance, whereas VTK.js supports multiple interpolation methods, including nearest neighbor and cubic.

The detailed description of the application’s architectural framework, the implementation via React and Cornerstone.js, and the experimental results on multiple platforms provide practical insights into the feasibility and effectiveness of the proposed solution. The study’s systematic approach, from design to implementation and evaluation, strengthens its contribution and applicability in real-world medical imaging scenarios.

### Limitations

This study focused on peripheral artery CT imaging for 22 patients and used a single dataset type. This dataset limitation may affect how broadly our findings can be applied to different medical imaging scenarios. Moreover, the application’s dependency on specific interpolation techniques for 3D reconstruction could limit its flexibility and efficiency in processing various types of medical imaging data. In addition, the application is designed to handle only DICOM formats, which may restrict its utility with other imaging formats prevalent in medicine. Furthermore, using vascular structures as reference points for comparing axial and reconstructed sagittal and coronal planes in the MPR can be challenging because of the uniformity of vascular structures, which often lack distinctive landmarks. This leads to inconsistencies in automatically produced reference lines and requires anatomical expertise for accurate validation.

### Comparison With Prior Work

A comprehensive test of our application was conducted using 2 distinct series from the same dataset, representing extremes in size. These series correspond to patients 2 and 17, as listed in Table 2. The dataset for patient 2 serves as a compact representation with dimensions of 512 × 512 × 5 pixels and a size of 2.51 MB, exemplifying the lower end of the size spectrum. Conversely, the dataset for patient 17 represents a substantial CT series with dimensions of 512 × 512 × 2339 pixels, occupying 1.10 GB, highlighting the challenges associated with handling voluminous medical image data. These series vary significantly in slice thickness, spacing between slices, and pixel spacing, ensuring a thorough evaluation of the application’s performance across diverse dataset sizes.

Highlighting the limitations encountered in the compared platforms, our work stands out as an innovative solution, as evidenced by the comprehensive performance analyses presented in Tables 6 and 7. In addressing compatibility concerns, our application surpassed competitors such as DicomViewer.net (version 3.2) [48], Image-IN (accessed February 2024) [25], BlueLight (accessed February 2024) [26], and VolView (accessed September 2024) [30]. DicomViewer.net, developed as an open-source project under the Open Health Imaging Foundation, is compatible with Google Chrome, Firefox, Safari, and Microsoft Edge, faced cross-browser compatibility issues on Firefox for macOS when dealing with both low- and large-size dataset series. Simultaneously, Image-IN, a web-based 3D visualizer for multidimensional DICOM microscopy images, encountered performance challenges on mobile devices, particularly iPads, leading to suboptimal performance and browser crashes with large dataset series. BlueLight is an open-source DICOM viewer with a low-cost computation algorithm but lacks security and maintenance considerations. In addition, the performance evaluation focused primarily on desktop browsers, and the compatibility and performance of mobile browsers or devices remain unclear. Addressing these limitations and conducting comprehensive evaluations across various platforms and scenarios would enhance the applicability and robustness of our proposed solution in real-world medical imaging contexts.

**Table 6:** Comparison of the performance of the proposed application with other applications over a local area network—low-size data.

Dataset, OS^a^ or tablet, and browser			Proposed software (seconds)				DicomViewer.net [48] (s)			Image-IN [25] : T1^b^+T2^c^ (s)		BlueLight [26] (s)			VolView [30]: T1+T2 (s)
			T1	T2	T1+T2	T1		T2			T1		T2		
**Dataset for patient 2**															
	**Windows**														
		Google Chrome	0.285	0.088	0.374	0.629		—^d^	1.63		0.448		0.325	1.586	
		Firefox	0.103	0.101	0.204	0.393		—	1.404		0.366		0.281	1.89	
	**Linux**														
		Google Chrome	0.125	0.095	0.22	0.45		—	1.869		0.388		0.305	1.262	
		Firefox	0.16	0.107	0.267	0.605		—	2.523		0.354		0.316	1.57	
	**macOS**														
		Google Chrome	0.228	0.115	0.343	0.421		—	2.144		0.569		0.454	3.32	
		Firefox	0.179	0.124	0.303	—		—	—		0.755		0.406	1.9	
	**Tablet**														
		Google Chrome	3.748	6.804	10.552	—		—	—		—		—	—	
		Firefox	4.752	7.576	12.328	2.416		—	—		1.578		1.826	—	

^a^OS: operating system.

^b^T1: performance time for loading a medical image dataset.

^c^T2: performance time to build a medical image volume using multiplanar reconstruction techniques.

^d^Crash or failure.

**Table 7:** Comparison of the performance of the proposed application with those of other platforms over a local area network—large-size data.

Dataset, OS^a^, and browser				Proposed software (seconds)							DicomViewer.net [48] (s)					Image-IN [25] : T1^b^+T2^c^ (s)			BlueLight [26] (s)					VolView [30]; T1+T2 (s)
				T1		T2		T1+T2		T1			T2					T1			T2			
**Dataset for patient 17**																								
	**Windows**																							
		Google Chrome	6.021		0.170		6.192		5.85			—^d^		—			310			9.623		—		
		Firefox	7.10		0.382		7.482		5.524			—		—			319			9.669		—		
	**Linux**																							
		Google Chrome	6.326		0.151		6.478		21.939			—		—			Stopped at slice 1750			—		—		
		Firefox	23.366		0.037		23.403		60.02			—		—			Stopped at slice 520			—		—		
	**macOS**																							
		Google Chrome	31.019		0.848		31.868		12.264			—		—			407			30.22		—		
		Firefox	16.628		0.024		16.652		—			—		—			—			—		—		

^a^OS: operating system.

^b^T1: performance time for loading a medical image dataset.

^c^T2: performance time to build a medical image volume using multiplanar reconstruction techniques.

^d^Crash or failure.

Table 6 shows that our application was significantly superior to DicomViewer.net, Image-IN, BlueLight, and VolView in terms of both loading times and reconstruction efficiency across various configurations. For example, when considering a low-size dataset series of 2.51 MB on Windows with Google Chrome, our application resulted in loading times (T1) and reconstruction times (T2) that were 63% to 85% faster than those of the competing platforms. Specifically, with Google Chrome on Windows, our application achieved a combined metric (T1+T2) of 0.374 seconds, whereas DicomViewer.net, Image-IN, BlueLight, and VolView experienced crashes or failures (shown as “—” in the table). Furthermore, when tested on a tablet, the proposed application outperformed the state of the art by maintaining a robust performance, whereas DicomViewer.net failed to build the MPR and both Image-IN and VolView were unable to upload and build the MPR. This reinforces the scalability and versatility of the proposed software across different device types. Table 7 shows that our application continued to outperform DicomViewer.net, Image-IN, BlueLight, and VolView by a significant margin in the analysis of large-size data of 1.10 GB, ranging from 84% to 98%. For example, when running on Linux with Google Chrome, our application achieved loading and reconstruction times that were notably faster than those of the competitors. However, BlueLight encountered issues and stopped at slice 1750 and at slice 520 for Linux on Google Chrome and Firefox, respectively. This suggests potential limitations in BlueLight’s ability to handle large dataset series, highlighting the robustness and scalability of our application. Similarly, VolView encountered a range error when processing a large dataset. Compared with DicomViewer.net, Image-IN, BlueLight, and VolView, our application consistently outperformed DicomViewer.net in terms of loading time and reconstruction efficiency, establishing it as a leading solution in medical image web visualization. The comparison reveals our application’s reliability and effectiveness in addressing the challenges encountered by existing platforms, making it a compelling choice for medical image visualization tasks. On the basis of the findings presented in Table 5, the average number of DICOM slices used for evaluating the application is 347. This evaluation was conducted within an LAN environment on a Windows platform.

**Figure 4:**
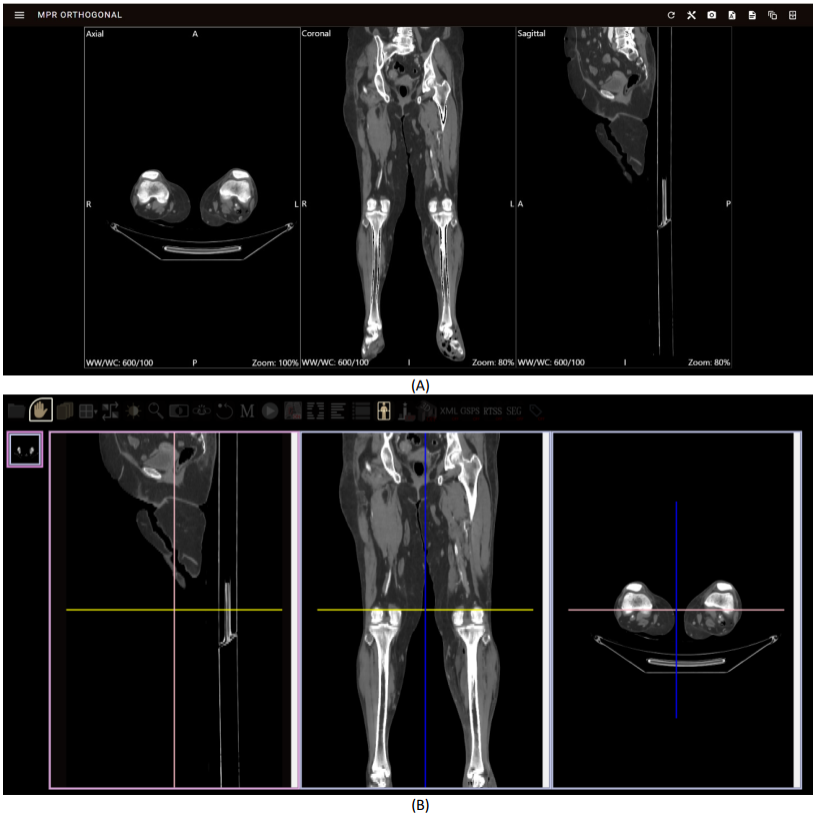
Comparison of Digital Imaging and Communications in Medicine and multiplanar visualization on the web (axial, coronal, and sagittal views) between (A) the proposed multiplanar reconstruction (MPR) method and (B) the BlueLight MPR result.

The combined performance time (T1+T2) ranged from 6.251 seconds when Google Chrome was used to 6.564 seconds with Microsoft Edge and 7.22 seconds with Firefox. In contrast, BlueLight, as reported in its corresponding study, used 280 DICOM slices for evaluation. Comparative analysis revealed that our application consistently exhibited shorter combined performance times across all the browsers, with durations of 8.91 seconds (Google Chrome), 9.15 seconds (Microsoft Edge), and 16.27 seconds (Firefox). Furthermore, as shown in Figure 4A, our proposed MPR algorithm leverages bicubic interpolation for edge pixels and weighted bilinear interpolation for nonedge pixels. This approach yielded favorable reconstruction results, particularly for edge pixels, compared with the MPR results produced by BlueLight, as illustrated in Figure 4B. These results underscore the superior performance of our application in loading medical image datasets and executing MPR techniques compared with BlueLight, thereby highlighting the efficiency and effectiveness of our application in processing medical image datasets and positioning it as a more dependable option for medical image visualization. On the basis of these results, our developed application achieved compatibility with all browsers and platforms, demonstrating accurate and fast processing. In addition, users can access and upload files or folders directly from their local computers, resulting in improved user interaction. These advancements surpass the findings of a previous study [18], which used HTML5 and WebGL for web-based medical imaging but encountered limitations such as compatibility issues with Internet Explorer, difficulties with user interaction for local file access, and reliance on predefined surface information for 3D visualization.

This study introduces PWA for DICOM and MPR visualization on the web, addressing challenges such as cross-platform compatibility, speed, and offline functionality. By leveraging PWAs, the application enhances accessibility and performance in medical imaging tasks, including offline access, which allows it to function without internet connectivity by caching essential resources. This is particularly useful in areas with limited connectivity. In addition, improved performance is achieved through React.js and IndexedDB (via Dexie), optimizing the handling of large datasets, reducing loading times, and accelerating MPR. These features enhance usability and efficiency, improving radiologists’ workflow.

Furthermore, our application outperforms existing platforms such as DicomViewer.net, Image-IN, and BlueLight in terms of loading time and reconstruction efficiency, positioning itself as a robust and reliable choice for medical image visualization.

### Conclusions

This study effectively addresses significant gaps in web-based medical imaging applications, particularly DICOM and MPR visualization via PWAs. Leveraging the unique features of PWAs, such as uninterrupted offline access and enhanced performance, substantial progress was made in overcoming technological barriers and advancing medical imaging functionality. Emphasizing cross-platform compatibility, integration capabilities, and speed underscores critical aspects in developing web-based medical imaging solutions. The proposed design and implementation demonstrate the feasibility and effectiveness of integrating DICOM and MPR visualization into web environments via PWAs, benefiting radiologists and health care professionals. Moreover, our study addressed MPR challenges, enhancing diagnostic capabilities through advanced interpolation methods and reconstruction protocols. The experimental results consistently showed superior performance compared with existing platforms, firmly establishing our application as a leading solution in medical image web visualization. The evaluation and testing were conducted using a dataset comprising CT scans from patients diagnosed with peripheral artery disease, adding real-world relevance and validation to our findings. Future work will focus on visualizing 3D surfaces and volume rendering via MPR images.

## Appendix

Multimedia Appendix 1 Control measurements of the distal edge of the L1 vertebra for patient 1 using 3D Slicer.

## Abbreviations

CT: computed tomography
DICOM: Digital Imaging and Communications in Medicine
LAN: local area network
MPR: multiplanar reconstruction
PACS: picture archiving and communication system
PWA: progressive web application
WAN: wide area network

## Data Availability

The dataset analyzed during this study are available from the corresponding author on reasonable request.
