# Supplementary material for: Advancing Progressive Web Applications to Leverage Medical Imaging for Visualization of Digital Imaging and Communications in Medicine and Multiplanar Reconstruction: Software Development and Validation Study
Source: JMIR Med Inform. 2024 Dec 9;12:e63834. doi: 10.2196/63834 (PMC11667143; doi:10.2196/63834)
Supplement: Multimedia Appendix 1 [file medinform_v12i1e63834_app1.docx]

| 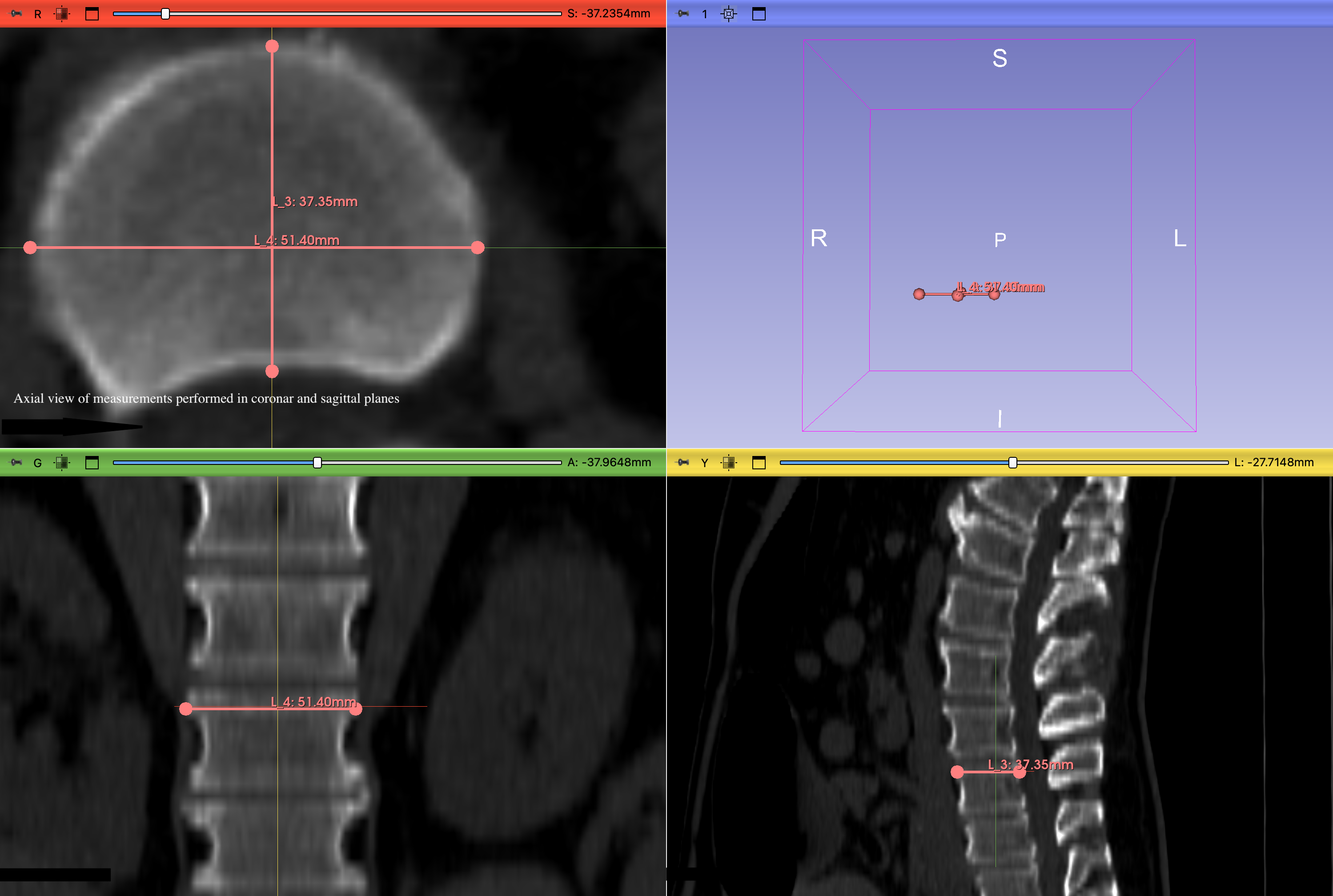 |
| --- |
| (a) |
| 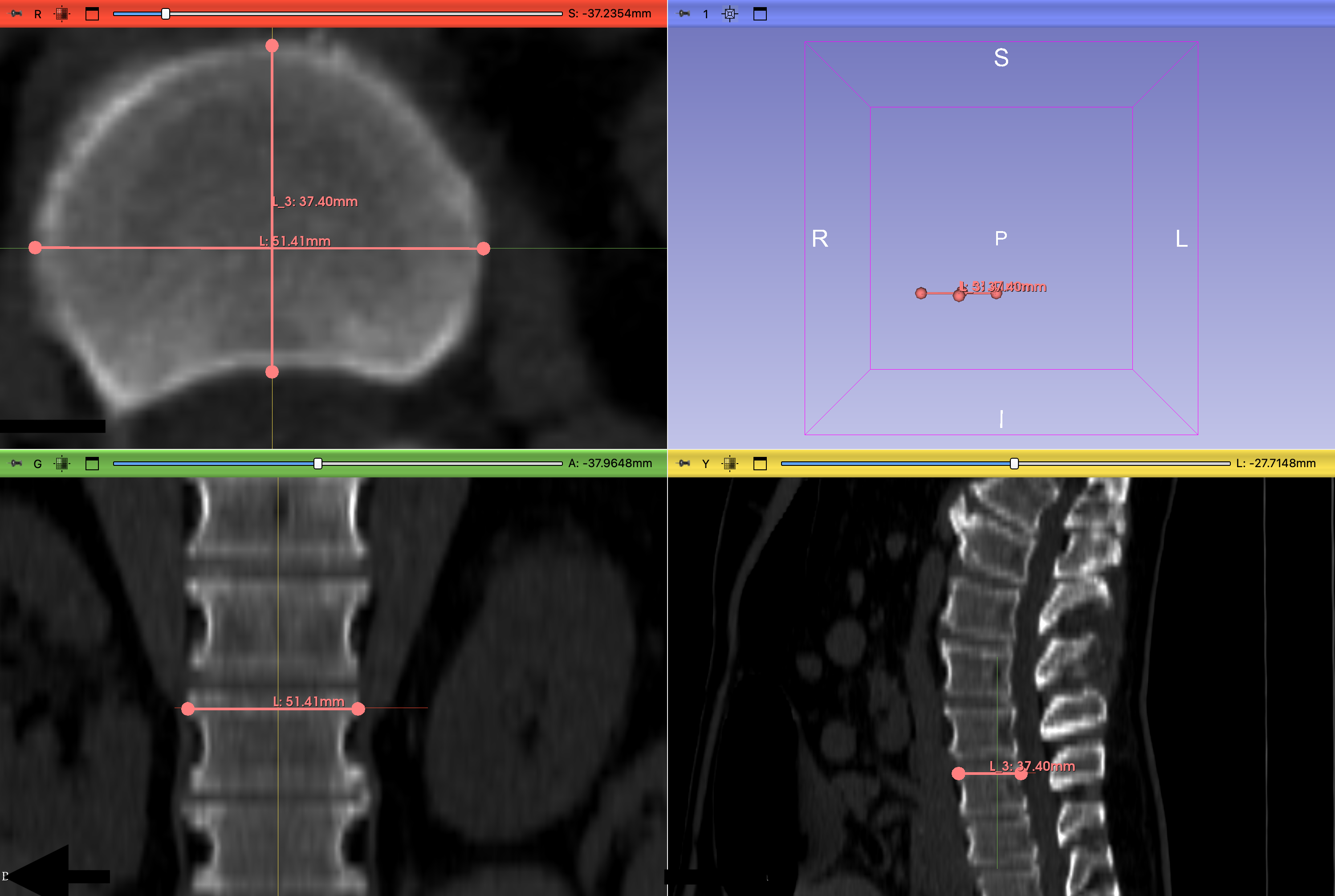 |
| (b) |

**Figure A1.** Control measurements of the distal edge of the L1 vertebra for patient #1 using 3D Slicer (Version 5.6.2). (a) Measurements performed in the axial view, automatically reflected in the coronal and sagittal planes. (b) Manual measurements repeated in the coronal and sagittal planes. Minor differences (<0.5mm) observed between the views are attributed to the manual measurement process using the mouse cursor.
